# Clinical commissioning of a novel compact multi‐room pencil beam scanning proton therapy system

**DOI:** 10.1002/acm2.70538

**Published:** 2026-03-15

**Authors:** Eunsin Lee, Austin M Faught, Hyeri A Lee, Estelle Batin, Ahmet Ayan

**Affiliations:** ^1^ Department of Radiation Oncology The Ohio State University Columbus Ohio USA

**Keywords:** PBS commissioning, ProBeam 360°, Proton

## Abstract

**Purpose:**

To present the clinical commissioning of the world's first multi‐room Varian ProBeam 360° proton pencil beam scanning system.

**Methods:**

The state‐of‐the‐art system includes two clinical treatment rooms with 360° rotating gantries, a superconducting cyclotron, an energy selection system, a beam transport system and scanning nozzles. These components deliver proton spots ranging from 3.97 to 30.03 g/cm^2^ to arbitrarily shaped target volumes over a scanning area of 25 cm × 25 cm at isocenter. Proton beam ranges (R_80_) were measured and verified independently in both gantry rooms to ensure agreement with beam specifications. Dosimetric parameters, including depth dose curves, in‐air spot profiles and dose per monitor unit (MU) as a function of energy, were measured and used to create a dose calculation model in the RayStation treatment planning system (TPS). Treatment plans with various sizes of spread‐out Bragg peaks (SOBPs) and simulated patient plans for a range of clinical sites were created and measured at various depths to validate the TPS's beam model accuracy. Additionally, beam matching between the two rooms was performed and validated.

**Results:**

Across the full energy range of 69–218 MeV, the measured R_80_ in both rooms were identical within 0.4 ± 0.2 mm deviation from the expected nominal range. In‐air spot sizes agreed within 5.7 ± 1.8% while outputs matched within 1.0 ± 0.5%. The average point dose difference was 0.2 ± 0.7% for over 80 measurements of SOBP validation plans from the TPS calculations and all planar dose measurements matched TPS calculations with over 90% of data passing a 2mm/2% gamma criterion. All patient plans were validated at a 3mm/3% criterion and demonstrated over 90% of data passing.

**Conclusion:**

Both gantry systems were successfully commissioned for clinical use. Accurate measurements with robust validation ensured essential parameters for beam delivery and dosimetry were characterized for safe patient treatments.

## 1 INTRODUCTION

The pencil beam scanning (PBS) proton beam delivery system^1^ is distinguished by its ability to modulate the intensity as well as the energy of the proton to deliver a homogeneous dose distribution tailored to the complex geometries of various cancers. The Varian ProBeam 360° system, utilizing PBS technology, is designed to enhance the clinical application of proton therapy by offering a compact, 360° rotating gantry‐room solution that is both cost‐effective and space‐efficient.

The original ProBeam system served as the backbone for most of Varian's multi‐room installations, featuring a gantry with a 10 m diameter, a field size of 40 × 30 cm^2^, and a more expansive beamline architecture compared to later designs. The ProBeam Compact was introduced as a single‐room solution, maintaining the same gantry and field size as the original ProBeam but reducing the overall footprint and installation complexity. The ProBeam 360° represents a further refinement—featuring a smaller gantry (8 m diameter), reduced field size (25 × 25 cm^2^), and a redesigned nozzle with modular beam transport components. These changes were originally developed for single‐room deployment but have now been adapted for multi‐room configurations. This adaptation required new validation workflows, regulatory clearance, and integration strategies not present in prior systems. The transition marks a significant advancement in mechanical efficiency, installation flexibility, and clinical throughput, distinguishing it from previous multi‐room ProBeam deployments.

The Ohio State University is the first site to install the state‐of‐the‐art PBS Varian Multi‐Room ProBeam 360° system. The successful commissioning of a first, newly installed system will provide valuable information on performance and limitations of the system for future centers utilizing this cutting‐edge technology. In addition, establishing of clinically acceptable beam‐matching across multi‐gantry rooms offers the advantage of reducing commissioning time and improving operational efficiency. This work may allow further investigation and validation of critical dosimetric parameters for potential use of a golden beam data set for beam matching across proton facilities with the same type of the system.

## 2 MATERIALS AND METHODS

### 2.1 ProBeam 360° overview

The ProBeam 360° introduces several structural and subsystem refinements compared to the original ProBeam,^2^, ^3^ in both cyclotron and nozzle design, reflecting their respective optimization goals. The ProBeam 360° employs the AC226 cyclotron,^4^ delivering a fixed maximum energy of 226 MeV with a maximum 800 nA current that can deliver a maximum range of 31.94 g/cm^2^, compared to the AC250 in ProBeam, which supports up to 250 MeV. The AC226 operates at a lower RF frequency (70.3 MHz vs. 72.8 MHz), with a central magnetic field of ∼2.4 T and a maximum coil field of ∼3.5 T, tuned for compactness and energy efficiency. It supports a dynamic current range of 1:800 and intensity modulation higher than 10% in 100 ms, with beam rotation at 35.15 MHz. This tuning supports a streamlined energy selection system (ESS) that relies on a double carbon wedge degrader that can reduce the clinical energy continuously^a^ from 218 to 69 MeV. In contrast, the ProBeam's higher energy output and broader modulation range are suited for larger field sizes and more extensive beamline configurations. For the nozzle, ProBeam 360° integrates compact, high‐speed scanning magnets (X: 300 kg, Y: 150 kg) with asymmetric source‐to‐axis distances (SAD X: 1340 mm, Y: 1750 mm), enabling scanning speeds up to 57.7 mm/ms. The nozzle also features a glass‐reinforced plastic vacuum chamber to minimize magnetic interference, a Kapton window to maintain vacuum integrity, a monitor unit (MU) chamber, and a multi‐strip ionization chamber for beam position verification. At isocenter, a maximum field size is 25 × 25 cm^2^—smaller than ProBeam's 40 × 30 cm^2^. Range shifters can be inserted into the snout, which can be extended from 42.1 cm to 4.0 cm from the isocenter. The gantry can rotate 10° past a full rotation in both clockwise and counterclockwise directions for a total of 380° of potential rotation in an International Electrotechnical Commissioning (IEC) 61217 coordinate system.^5^ The treatment couch allows rotation between 260° and 100°, with 0° defined as the nominal position, for a total of 200° of possible rotation. Clinically, pitch and yaw adjustments of up to ±3° are allowed. The planned energy layer switching time is less than 200 ms, and the minimum time required to deliver the lowest weighted spot per energy layer is approximately 2 ms. A typical nozzle current for patient treatment is 0.5–1 nA. The dose rate is determined by the spot with the lowest number of MUs per layer. A deliverable minimum MU per spot is set to 2 MU.

### 2.2 Beam characterization

Report of AAPM Task Group 185 is served as a guideline for required measurements, technique and equipment for commissioning beam data for PBS system.^6^ The RayStation TPS (RaySearch Laboratories, Stockholm, Sweden) is adopted to model PBS proton treatment planning. The beam data required for developing a model in RayStation, such as ranges, in‐air spot profiles, and output factors were acquired. These fundamental properties of proton pencil beams were characterized over its full clinical energy range of 69 to 218 MeV.

#### 2.2.1 Integrated depth dose (IDD) curves

IDD profiles were obtained by measuring pristine Bragg peaks (BPs) in a 3D water phantom (MP3‐PL, PTW‐Freiburg, Germany) using a large area of 4.1 cm radius BP chamber (Bragg Peak Chamber Type 34070, PTW‐Freiburg, Germany) and a thin window reference chamber (Bragg Peak Chamber Type 34080, PTW‐Freiburg, Germany). Measurements were acquired in 5 MeV increments from 70 to 215 MeV with additional measurements performed at the maximum and minimum energies of 69 and 218 MeV. For all beam energies, the measured BP ranges (R_80_, the depth of the distal 80% of the maximum dose value of the BP in water) were compared to the National Institute of Standards and Technology (NIST) published stopping power and range tables for proton energies (PSTAR).^7^ The NIST PSTAR table is adopted and used to define the nominal values of proton beam energies by Varian. Ranges were acquired at gantry angles of 0° and 90°.

#### 2.2.2 Spot size and virtual source position

In‐air spot profiles were measured using Lynx (IBA Dosimetry, Schwarzenbruck, Germany) scintillation‐based imager in 5 MeV energy step from 70 to 215 MeV with additional measurements at 69 and 218 MeV. RayStation TPS requires in‐air spot profile measurements at least three different depths to model the virtual source position and divergence of the beam and recommends two additional depths relative to isocenter: −15, −10, 0, 10, 20 cm, where −15 cm was chosen due to the vertical limits of the robot couch supporting the measurement device. Following AAPM TG‐185 guidance to account for gantry‐angle dependency, we acquired spot profiles at 0° and 90°, which bracket the principal sag and alignment conditions of scanning magnets for a rotating gantry^b^. These angles capture the largest expected variations within vendor tolerances that specify spot size variations within ±10% across angles. Therefore, the measurements were performed at gantry angles of 0° and 90°. The average of *x*‐ and *y*‐profiles, acquired at both gantry angles, were used to form a single spot profile per energy for use in the TPS. Virtual source‐to‐axis distances (SAD) were calculated using the measurements of four different spot locations at −12.0 and 12 cm from the center along *x*‐ and *y*‐axis, as defined at isocenter, at two different depths. The divergence from virtual source position was compared with the expected values from the treatment delivery system vendor specification.

#### 2.2.3 Absolute dose per MU

The dose per MU relationship was established using the methodology recommended by the International Atomic Energy Agency (IAEA) TRS‐398 protocol for determination of absorbed dose from proton beams.^8^ Individual measurements were performed using a uniform, single energy‐layer field for the full clinical energy range in 5 MeV steps. The 10 × 10 cm^2^ fields were created with spots arranged in a rectangular grid with uniform spacing of 2 mm. All spots have 5 MU per spot assigned to create a uniform lateral profile. An Accredited Dosimetry Calibration Laboratory (ADCL) calibrated plane‐parallel ionization chamber (Advanced Markus Chamber Type 34045, PTW‐Freiburg, Germany) associated with an electrometer (UNIDOS Romeo, PTW‐Freiburg, Germany) were used to measure the absorbed dose in water. The effective point of measurement was placed at isocenter with a depth of 2 cm in water for all the energies, and plans were delivered at a gantry angle of 0°. Gantry angle dependency of output was validated with output measurements in solid water at four cardinal angles using a cylindrical ion chamber. The measured physical doses were entered into the RayStation TPS and the relative biological effectiveness (RBE) model with a constant factor 1.1 was applied to the physical dose at the time of dose calculation.

### 2.3 Treatment planning system (TPS) beam modeling and validation

#### 2.3.1 TPS beam modeling

Beam model creation in the RayStation TPS (version 12ASP1) requires measured pristine BPs, in‐air spot profiles, and absolute dose. The absolute dose measurement was performed at a depth in water, 2 cm, where the dose gradient is minimal along the depth axis. The energies of the absolute dose, the spot profile and pristine BP measurements must cover the minimum and maximum energy supported by the machine, in this case from 69 through 218 MeV. RayStation explicitly models the use of range shifters by considering the physical thickness, material composition, density, and geometry with respect to the nozzle, snout, and treatment isocenter. As a result, beam data acquired with range shifters is not required for the building of a model within the TPS.

#### 2.3.2 CT calibration

Two Siemens computed tomography (CT) scanners, Somatom Go.Open Pro and Somatom Drive (Siemens Medical Solutions, USA), were installed at the Ohio State University proton therapy center. CT calibration curves mapping Hounsfeld Units (HU) to mass density were generated following a consensus guideline^9^ that was created in a joint effort between the European Society for Radiotherapy and Oncology (ESTRO) Physics Workshop 2021 and the European Particle Therapy Network (EPTN) Work Package 5 (WP5). The Gammex Advanced Electron Density (AED) Phantom (Sun Nuclear Corporation, USA) with tissue equivalent plugs was scanned to establish the HU‐to‐density table. Additional high density metal inserts (aluminum, titanium and stainless steel) were added to characterize for potential metal implants. Since RayStation uses an internal mass density‐to‐stopping power ratio conversion, TPS predicted values of the relative linear stopping power (RLSP) for both CT scanners were compared with a published literature^10^ and the Imaging and Radiation Oncology Core‐Houston (IROC‐H) phantom test of HU to RLSP conversion curve.

#### 2.3.3 Validation of beam model

The TPS beam model was validated through a series of measurements designed to test core components of the beam model (e.g., range) in increasingly challenging geometries. Absolute dosimetry was verified using single energy layer plans. Plans for every 10 MeV provided direct comparison data to validate the model against the measurements used to create the model, while a systematic shift of those plans by 3 MeV created an additional, independent validation set not used for the creation of the model. Point doses were extracted at a depth of 2.0 cm water equivalent thickness (WET) and compared to measurements performed with an Advanced Markus chamber placed at an equivalent depth in solid water.

Spread out Bragg peak (SOBP) plans were also created with varying ranges, modulations, and field sizes for range, point dose, and two‐dimensional planar comparisons. Point dose comparisons were performed using measurements acquired with an Advanced Markus chamber positioned at the center of the SOBP. As means of comparing calculated range in the TPS to measurements, each of the SOBP plans was delivered to the Zebra multi‐layer ionization chamber (IBA Dosimetry, USA). The depth of the distal R_90_, R_80_, and R_20_ were compared between measurements and the TPS. A subset of the SOBP plans were measured with the MatriXX PT 2D ionization chamber array (IBA Dosimetry, USA) at the effective planes of measurement equal to the center of the SOBP, and ¼ and ¾ distances within the SOBP. Comparisons were performed using the gamma index^11^ with a criterion of ±2%/2mm with a 10% threshold.

A similar methodology to the open field validation measurements was used to test the TPS modeling of range shifters. Two additional parameters were used in creating fields and evaluating the robustness of the modeling: range shifter thickness (2, 3, and 5 cm physical thickness) and the air gap between the range shifter and solid water surface. Air gaps tested ranged from 5 cm up to 36.1 cm. Beyond absolute dose comparisons, 2D dose array comparisons, and SOBP IDD comparisons, the WET of each range shifter was compared between Zebra measurements and RayStation generated plans.

Mock clinical treatment plans were created on prior patient CT scans, and patient specific quality assurance (PSQA) was performed as a test of beam model accuracy. Treatment plans were chosen to span a variety of clinical sites likely to be treated and to utilize different range shifter thicknesses. Planar dose comparisons were made at two depths per field for eight different simulated treatment plans. Treatment plans included the following anatomic and disease sites: craniospinal irradiation, head and neck, esophagus, lung and brain. A total of 56 planar dose comparisons were made between MatriXX PT measurements and TPS calculated doses. Among all the fields used for the comparisons, each commissioned range shifter was used at least once. PSQA measurements were performed with the IBA MatriXX PT and analysis utilized the gamma index with a criterion of ±3%/3 mm with a 10% threshold. Plans were also analyzed with ±2%/2 mm and ±3%/2 mm gamma criteria to better understand model limitations.

#### 2.3.4 Beam matching: Statistical analysis

Beam matching between gantry rooms was evaluated using paired comparisons of absolute dose (cGy/MU), in‐air spot size σ (mm) in x and y, and distal range R_80_ (mm) across matched energies. We report the mean difference between gantry‐room (GR) 1 and 2, 95% confidence intervals (CI), and paired *t*‐test *p*‐values. Clinical equivalence was assessed using the two one‐sided tests (TOST) procedure, which determines whether the true difference lies within a pre‐specified margins: dose ±1.5% (more stringent than 1.7% of the combined uncertainty in reference proton dosimetry^8^), R_80_ ±1 mm, and spot size ±10% (vendor tolerance). For PSQA, paired comparisons of gamma pass rates at 3%/3 mm, 3%/2 mm, and 2%/2 mm were analyzed as percentage‐point (pp) differences with corresponding TOST margins to ±2 pp for all criteria, reflecting clinically acceptable variability and aligning with reproducibility range for PSQA. Statistical significance was set to α  =  0.05 as the decision threshold, ensuring an overall 95% confidence that the true difference lies within the equivalence margin and that any observed difference is clinically negligible.

#### 2.3.5 Secondary dose calculation implementation and validation

To develop a secondary dose calculation tool, the Monte Carlo code MCsquare (MC2)^12^ was utilized as a part of an automated workflow. Implementation of the tool included CT calibration, beam modeling, model validation and clinical workflow setup. MC2 beam modeling used the commissioning beam measurement data. To validate the beam model, IDDs, absolute dose and in‐air spot profiles including with range shifters were simulated and ten clinical cases were compared to measurements data. For clinical workflow, a background script monitors newly dispatched jobs and initiates the MC calculation when present. A report is generated including a point dose comparison and 3D gamma analysis with ±2%/2 mm to fit clinical guidelines and billing requirements.

## 3 RESULTS

### 3.1 Beam characterization and calibration

#### 3.1.1 IDD curves

**FIGURE 1:**
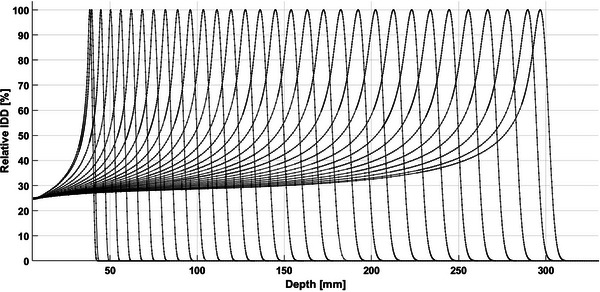
Relative IDD measurements normalized to max dose for the energies of 69–218 MeV at GR1.

**FIGURE 2:**
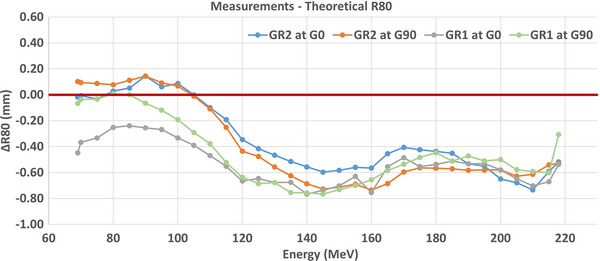
The measured range (distal R80) is presented for two different gantry rooms and at the two different gantry angles for each room.

IDD measurements in water acquired at a gantry angle of 0° in GR1 for the energies of 69–218 MeV in increments of 5 MeV are shown in Figure 1. Table 1 shows that the measured R_80_ in both GR1 and GR2 are within 0.4 ± 0.2 mm deviation from the theoretical values from NIST PSTAR. The measured ranges in both rooms match within 0.2 ± 0.1 mm over the full range of clinically available energies. Range constancy at two different gantry angles in each treatment gantry room is shown in Figure 2. The measured range differences between gantry angle of 0° and 90° in each room are 0.1 ± 0.1 and 0.0 ± 0.1 for GR1 and GR2, respectively.

**TABLE 1:** Measured and theoretical R80 and comparisons.

Energy (MeV)	Theoretical R80 (mm)	R80 measured at GR1 (mm)	R80 measured at GR2 (mm)	R80 at GR1 difference from theoretical (mm)	R80 at GR2 difference from theoretical (mm)	R80 at GR2 difference from GR1 (mm)
69	39.7	39.3	39.7	−0.4	0.0	0.4
70	40.7	40.4	40.7	−0.4	0.0	0.4
75	46.1	45.8	46.1	−0.3	0.0	0.3
80	51.8	51.5	51.8	−0.3	0.0	0.3
85	57.7	57.5	57.7	−0.2	0.1	0.3
90	63.9	63.6	64.0	−0.3	0.1	0.4
95	70.3	70.1	70.4	−0.3	0.1	0.3
100	77.1	76.7	77.2	−0.3	0.1	0.4
105	84.1	83.7	84.1	−0.4	0.0	0.4
110	91.3	90.8	91.2	−0.5	−0.1	0.4
115	98.8	98.2	98.6	−0.6	−0.2	0.4
120	106.5	105.8	106.1	−0.7	−0.3	0.3
125	114.4	113.8	114.0	−0.6	−0.4	0.2
130	122.6	121.9	122.1	−0.7	−0.5	0.2
135	131.0	130.3	130.5	−0.7	−0.5	0.2
140	139.6	138.9	139.1	−0.8	−0.6	0.2
145	148.5	147.8	147.9	−0.7	−0.6	0.1
150	157.6	156.9	157.0	−0.7	−0.6	0.1
155	166.8	166.2	166.3	−0.6	−0.6	0.1
160	176.3	175.6	175.8	−0.8	−0.6	0.2
165	186.0	185.5	185.6	−0.6	−0.5	0.1
170	195.9	195.4	195.5	−0.5	−0.4	0.1
175	206.0	205.4	205.6	−0.6	−0.4	0.1
180	216.3	215.7	215.8	−0.5	−0.4	0.1
185	226.7	226.2	226.3	−0.5	−0.5	0.1
190	237.4	236.9	236.9	−0.5	−0.5	0.0
195	248.3	247.7	247.7	−0.5	−0.6	0.0
200	259.3	258.7	258.6	−0.6	−0.6	−0.1
205	270.5	269.8	269.8	−0.6	−0.7	0.0
210	281.9	281.2	281.1	−0.7	−0.7	0.0
215	293.4	292.8	292.8	−0.7	−0.6	0.1
218	300.4	299.9	299.9	−0.5	−0.5	0.0

#### 3.1.2 Spot size and virtual source distance

**FIGURE 3:**
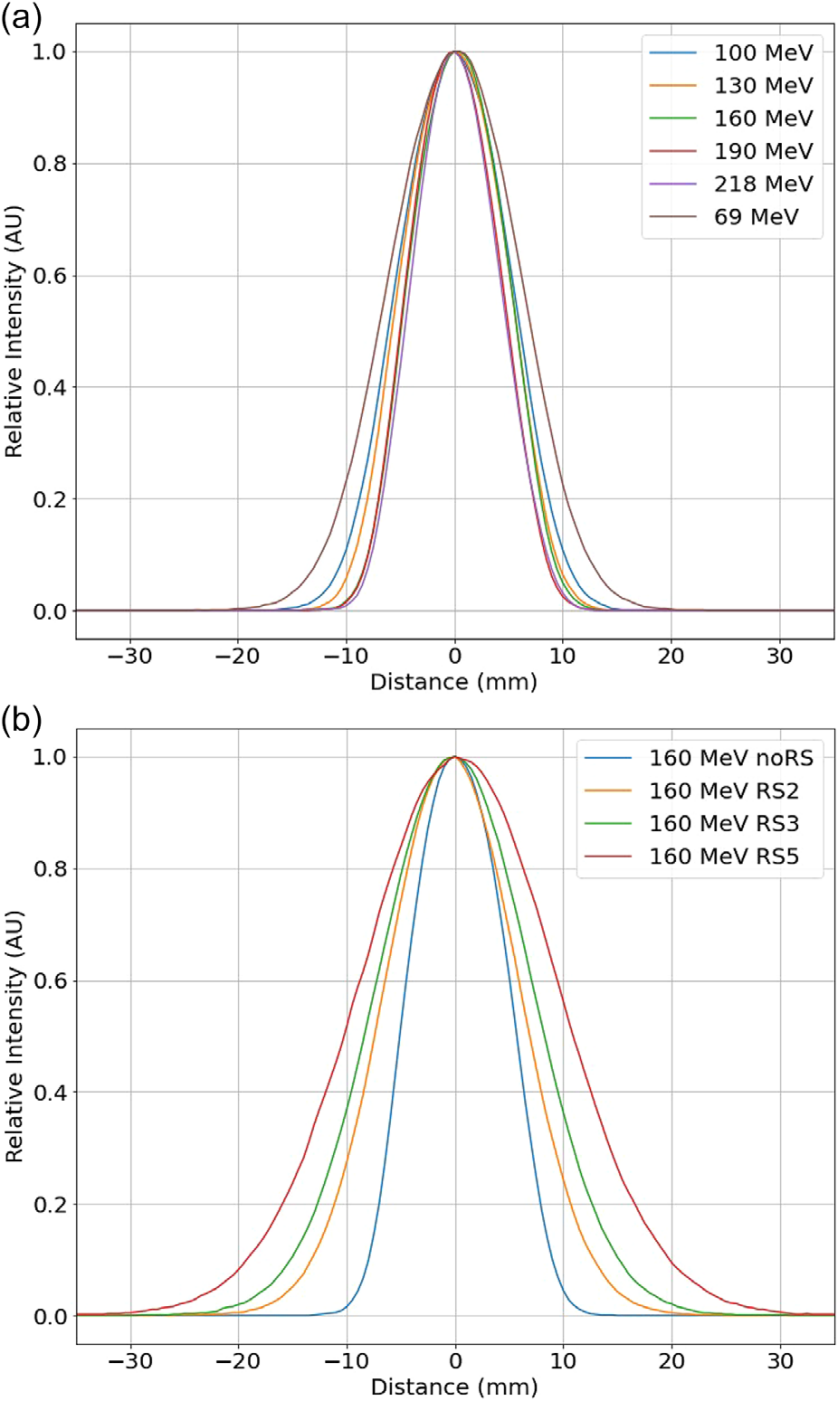
(a) In‐air spot profiles at isocenter for six different energies and (b) with various range shifters for 160 MeV.

**FIGURE 4:**
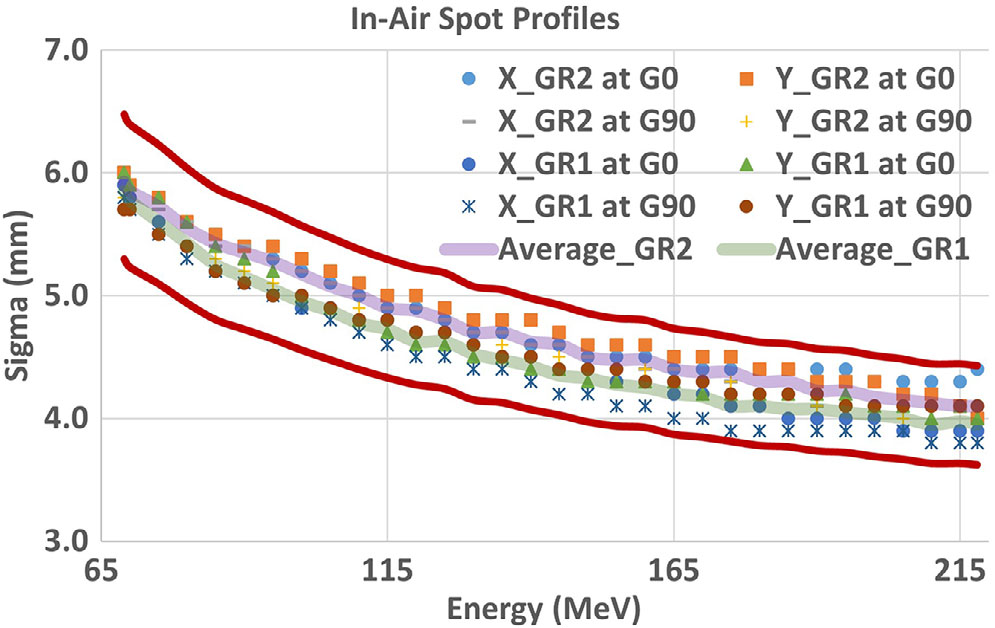
The values of σx and σy from a single Gaussian fit to in‐air spot profile measurements at isocenter as a function of energy are displayed for gantry angles 0° and 90° for both rooms. All data were within ±10% tolerance (red solid line) provided by Varian.

Figure 3a shows in‐air spot profiles without range shifter measured at the isocenter in GR1 for 6 representative energies of 69, 100, 130, 160, 190 and 218 MeV. The in‐air spot profile measurements with 2, 3 and 5 cm range shifters at the isocenter for 160 MeV are shown in Figure 3b. All measurements for the full range of energies were fitted with a single gaussian in each x and y direction at both gantry angles of 0° and 90°. In Figure 4, the values of σ_x_ and σ_y_ are shown as a function of energy at each gantry angle in both rooms. All values of σ are within the tolerance of ±10% provided by the treatment delivery system manufacturer. The average of *x*‐ and *y*‐profiles acquired at 0° and 90° were used for the TPS beam modeling.

The virtual source distances for *x*‐ and *y*‐scanning magnets were measured as 1335.9 (1136.9) mm and 1751.9 (1753.5) mm for GR1 (GR2) and verified to be within 0.3% agreement with the manufacturer specified values of 1340 and 1750 mm.

#### 3.1.3 Output factors

**FIGURE 5:**
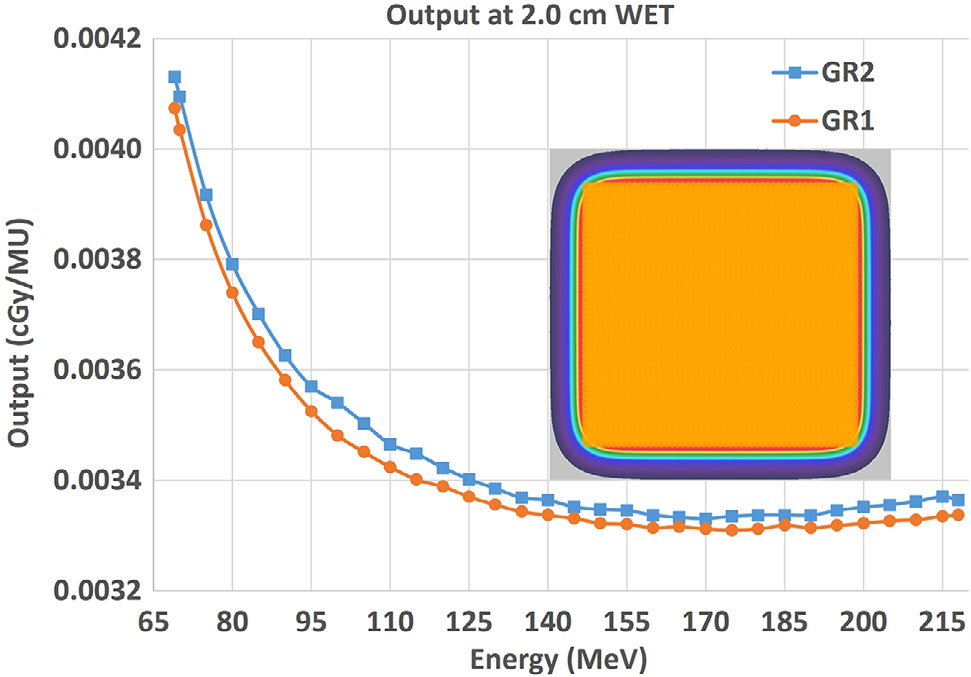
The absolute dose output measured as a function of energy for GR1 and GR2 using 10 × 10 cm^2^ single energy layer uniform fields.

Absolute dose per MU, as a function of energy, for GR1 and GR2 are shown in Figure 5. The absolute doses for each gantry were measured within 1.0 ± 0.5% with a maximum difference of 1.7%. A systematic trend of higher dose measured in the second treatment room (GR2) was observed. The absorbed doses in water were reported as physical dose in the unit of cGy. The corresponding TPS calculated doses were ∼1% higher for each room. It was decided that to split the difference in an effort to use a common beam model for both rooms, the absolute dose measurements from GR1 would be used for the TPS modeling.

### 3.2 TPS beam modeling and validation

#### 3.2.1 CT calibration curve determination and RLSP

**FIGURE 6:**
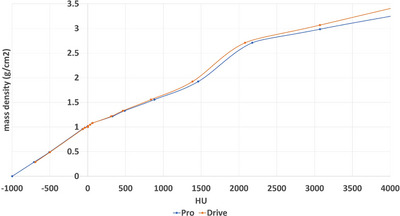
HU calibration curves for two Siemens scanners (Somatom Go.Open Pro and Somatom Drive) are presented.

Figure 6 shows the CT calibration curves for each scanner averaged over 7 different protocols (brain, head and neck, chest, abdomen and pelvis for adult patients and brain and abdomen for pediatric patients). The HU differences within different protocols and phantom sizes were clinically negligible and an average calibration curve was utilized for each scanner.

Table 2 provides the HU differences for high Z materials between two insert locations (center minus periphery) when the larger phantom was used (body size). Measurements were performed following the European consensus guideline.^9^ The differences were within 2% of the respective metal's scaled CT number for both scanners.

**TABLE 2:** HU value differences for high Z materials between two insert locations (center minus periphery) are presented.

Insert/Scanner	Open.Pro	Drive
HE Inner Bone	10.80	20.12
CaCO_3_ 30%	13.61	24.33
CaCO_3_ 50%	23.08	30.65
HE Cortical Bone	29.54	37.06

The result of IROC‐H phantom test of HU to RLSP conversion curve for the Somatom Go.Open Pro and the Somatom Drive CTs agree within 13% and 2.3%, respectively, where the IROC‐H's agreement criterion is ±15%.

#### 3.2.2 TPS beam model validation

##### Single layer absolute dose

Point dose measurements were performed in GR2 and comparisons validating the absolute dose accuracy of the TPS beam model showed a mean agreement of −1.0% with a range from −2.4% to −0.5%. All but the two lowest energies tested, 70 MeV and 73 MeV, showed agreement within ±2.0%. Overall, the predicted dose showed a systematic trend toward underpredicting the measured dose. Gantry angle dependency of output were agreed within 0.13% at 0°, 90°, 180° and 270° for both treatment rooms.

##### SOBP comparisons

Plans of SOBPs consisted of field sizes ranging from 1 × 1 cm^2^ up to the maximum field size of 25 × 25 cm^2^. Distal ranges of the SOBPs extended from 30 cm in water to 6 cm, and the modulations spanned 20 cm to 3 cm. Point dose comparisons at the center of an SOBP showed a mean agreement of 0.3 (0.5) % with range of −1.1 (−1.0)% to 1.8 (2.4)% for GR1 (GR2). The distal R_90_, R_80_, and R_20_ of the SOBP fields agreed with measurements to within 1.3 (1.9), 1.2 (1.8), and 1.5 (1.7) mm, respectively for GR1 (GR2). Average agreement for the three distal ranges was 0.1 (0.8), 0.1 (0.7), and 0.0 (0.6) mm, respectively for GR1 (GR2). Planar dose comparisons using gamma analysis passed a 2%/2 mm criterion at rates above 90% for all plans, and the average passing rate was 97.9 (98.6)% for GR1 (GR2).

##### Range shifter validation

Range shifters of physical thickness 2 cm, 3 cm, and 5 cm were commissioned in the TPS. The measured WETs of the three range shifters were 2.2 cm, 3.3 cm, and 5.6 cm, respectively. TPS calculated WETs were 2.3 cm, 3.4 cm, and 5.7 cm, respectively. Overall agreement was within 0.1 cm for all three range shifters commissioned.

When comparing dose measurements at the center of an SOBP with a range shifter in place, the average agreement was 0.4 (0.1)% with a maximum difference between measurement and TPS calculated dose of 1.9 (1.9)% corresponding to a TPS calculated dose lower than measurement for GR1 (GR2).

Zebra measurements of SOBP plans with range shifters in place were compared to the TPS calculated range. The maximum differences observed were 1.2 (0.8) mm, 1.2 (1.1) mm, and 1.0 (1.0) mm for R_90_, R_80_, and R_20_, respectively for GR1 (GR2). Average differences across all fields were 0.7 (0.5) mm, 0.7 (0.6) mm, and 0.7 (0.7) mm for R_90_, R_80_, and R_20_, respectively for GR1 (GR2).

Planar dose comparisons utilizing the gamma index with a criterion of ±2%/2 mm was performed for the SOBP plans for GR2 and spot‐checked for GR1, and the average passing rate was 98.9%. The minimum passing rate (range 6 cm, modulation 3 cm, field size 20 × 20 cm^2^ and 2 cm range shifter) was 88.7%, and all other fields had a passing rate above 94%. When evaluated at ±3%/2 mm, the minimum passing rate increased to 99.8%, indicating that the absolute dose measured by the MatriXX PT was right at the ±2% part of the more restrictive gamma criteria.

##### Mock clinical plans: Patient specific QA

**FIGURE 7:**
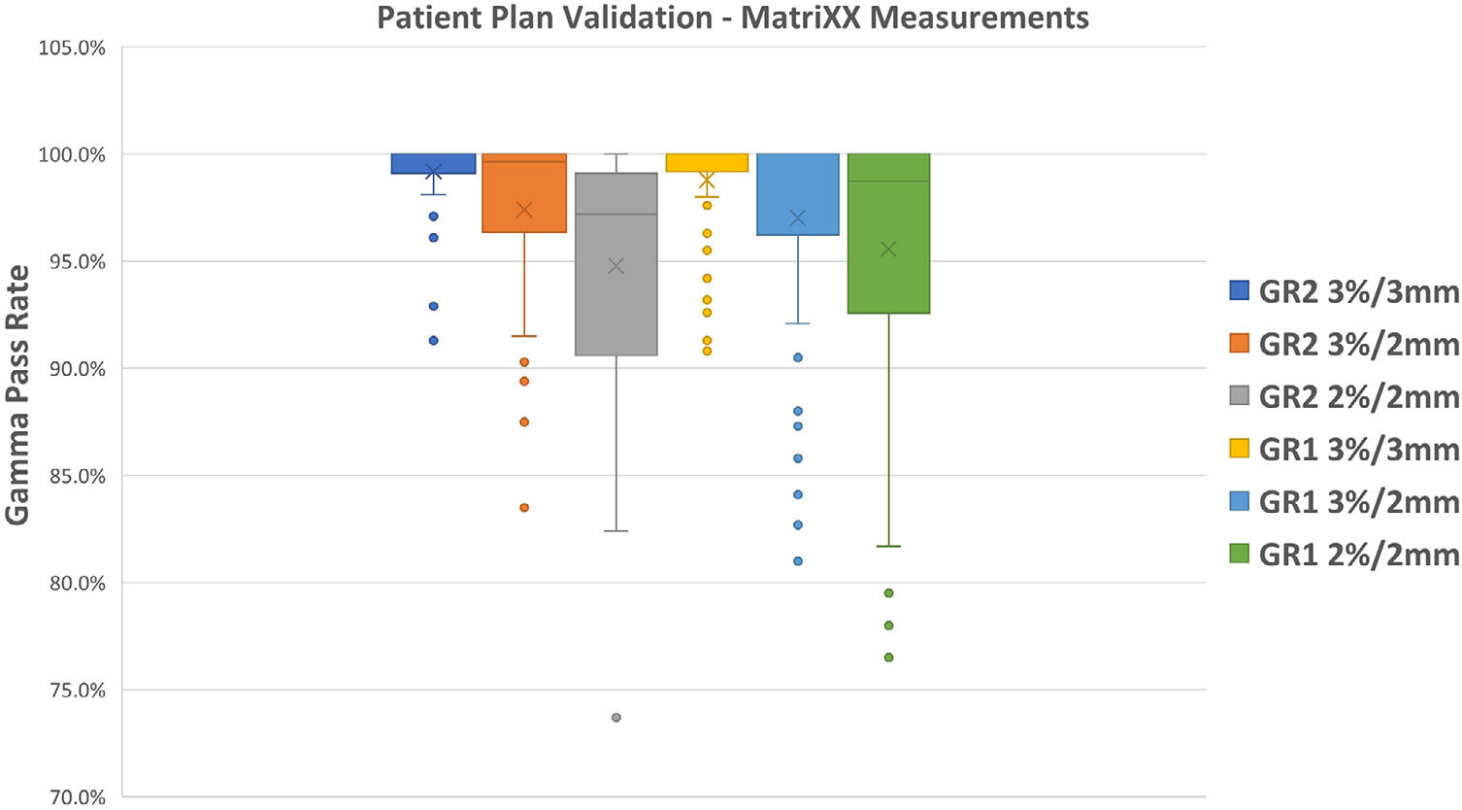
A box and whisker plot of the pass rates planar dose comparisons for simulated clinical plans is presented for three different gamma criteria.

Dose comparisons were performed with gamma criteria of ±3%/3 mm, ±3%/2 mm, and ±2%/2 mm (Figure 7). The average passing rates for the three criteria were 98.8% (99.2%), 97.0% (97.4%), and 95.6% (94.8%) for GR1 (GR2), respectively. All comparisons exhibited greater than 90% of pixels passing the ±3%/3 mm criteria (Table 3).

**TABLE 3:** A summary of the passing rates for planar dose comparisons between treatment planning system calculated doses in simulated clinical plans and measurements conducted with the MatriXX PT system is presented. Gamma criteria of ±3%3 mm, ±3%/2 mm, and ±2%/2 mm were all used.

	Passing rate statistics						Fraction of plans with passing rates			
	Average		Median		Minimum		90%		95%	
Gamma criteria	GR1	GR2	GR1	GR2	GR1	GR2	GR1	GR2	GR1	GR2
3%/3mm	98.8%	99.2%	100%	100%	90.8%	91.3%	100%	100.0%	87.5%	96.4%
3%/2mm	97.0%	97.4%	100%	99.7%	81.0%	83.5%	89.3%	91.1%	78.6%	80.4%
2%/2mm	95.6%	94.8%	98.8%	97.2%	76.5%	73.7%	80.4%	76.8%	71.4%	64.3%

#### 3.2.3 Beam matching summary

##### Fundamental beam parameter comparisons

Across 69–218 MeV, the mean GR2–GR1 difference was +0.99% for absolute dose (95% CI: 0.87–1.11%), +0.19 mm for R_80_ (95% CI: 0.13–0.24 mm), +6.08% for σ_x_ (95% CI: 5.35–6.81%), and +2.23% for σ_y_ (95% CI: 1.53–2.93%). R_80_, spot sizes and absolute dose met equivalence criteria of ±1 mm, ±10% and ±1.5%, respectively. Extrema across energies were 0.49%–1.69% for absolute dose, 1.71%–10.39% for σ_x_ and −2.47%–4.76% for σ_y_, and −0.07–0.43 mm for R_80_ (Table 4).

**TABLE 4:** Paired statistical analysis of beam parameters between gantry rooms (GR2–GR1). Mean differences, 95% confidence intervals (CI), and paired *t*‐test *p*‐values are reported. Clinical equivalence using two one‐sided tests (TOST) against pre‐specified margins: absolute dose ±1.5%, range ±1 mm, and spot size ±10%.

Parameter	N	Mean difference	95% CI	Paired *p*	Equivalence margin	TOST
Absolute dose	32	+0.99%	0.87–1.11%	< 0.001	±1.5%	Equivalent
R_80_	32	+0.19 mm	0.13–0.24 mm	< 0.001	±1.0 mm	Equivalent
Spot σ_x_	32	+6.08%	5.35–6.81%	< 0.001	±10%	Equivalent
Spot σ_u_	32	+2.23%	1.53–2.93%	< 0.001	±10%	Equivalent

##### Patient specific QA comparisons

For a total of 56 planar doses from eight simulated plans across lung, HN, esophagus, CNS, CSI, paired comparisons (GR2–GR1) yielded mean percentage‑point differences of +0.40 pp (3%/3 mm), +0.38 pp (3%/2 mm), and −0.78 pp (2%/2 mm). All three criteria met equivalence within pre‑specified margin of ±2 pp (Table 5).

**TABLE 5:** Paired comparison of PSQA gamma pass rates between gantry rooms (GR2–GR1) for three criteria. Differences are expressed in percentage points (pp) with 95% CI and paired *t*‐test *p*‐values. Equivalence was evaluated using TOST against margins of ±2 pp. All criteria met equivalence, supporting consistent PSQA performance across rooms.

Parameter	N	Mean difference	95% CI	Paired *p*	Equivalence margin	TOST
3%/3 mm	56	+0.40 pp	−0.01–0.80 pp	0.053	±2 pp	Equivalent
3%/2 mm	56	+0.38 pp	−0.14–0.91 pp	0.146	±2 pp	Equivalent
2%/2 mm	56	−0.78 pp	−1.90–0.33 pp	0.165	±2 pp	Equivalent

#### 3.2.4 Secondary dose calculation validation

**FIGURE 8:**
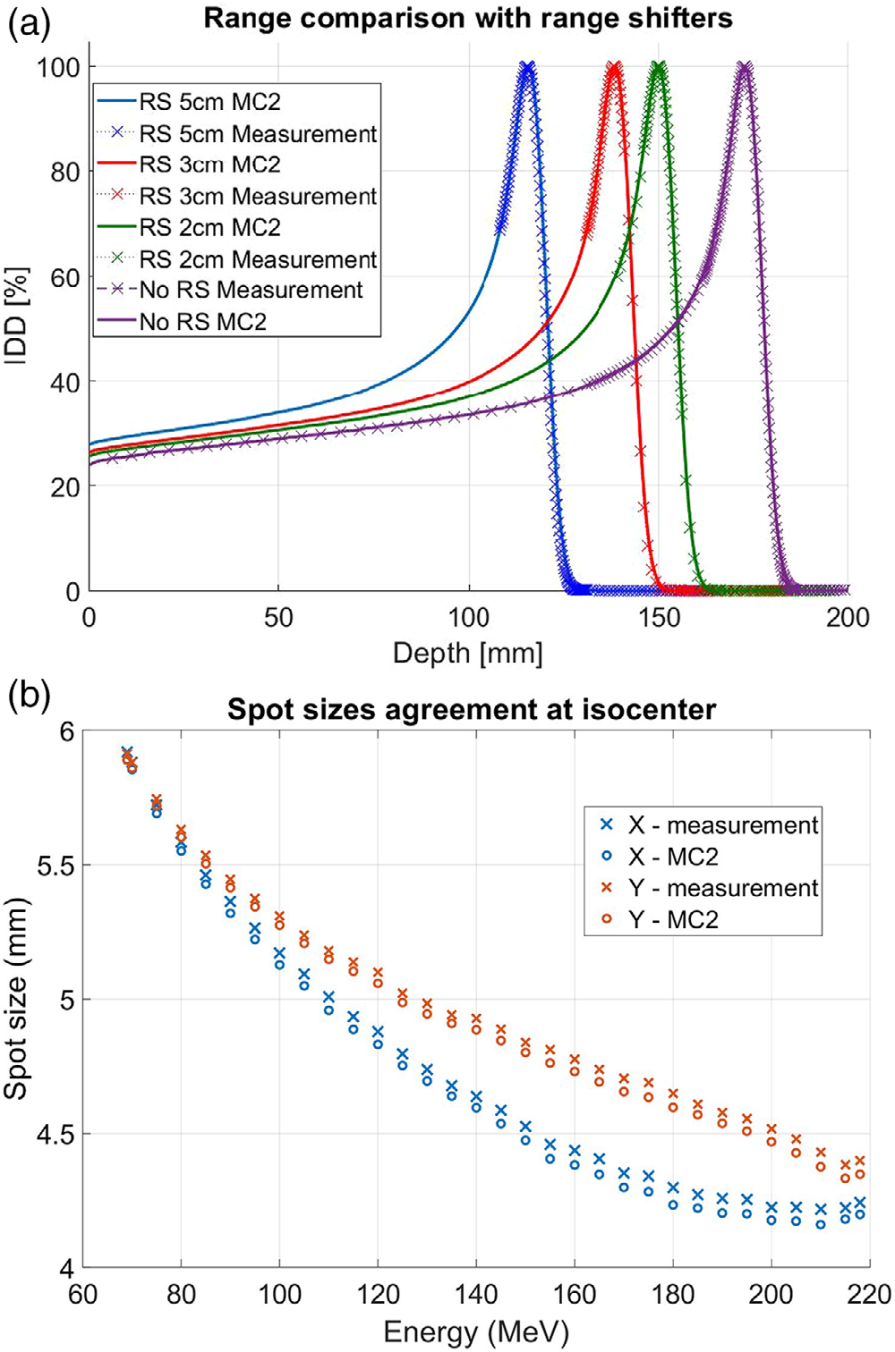
(a) IDD comparison between the commissioning measurements and MC2 predictions for 160 MeV energy pristine peak without range shifter and with 2, 3 and 5 cm range shifters and (b) In‐air spot size comparison between commissioning measurements and MC2 predictions at the isocenter.

The calculated ranges from the secondary dose calculation algorithm were compared to measurements without range shifters (Figure 8a). Good agreement was found for the full range of clinical energies (69–218 MeV) with 0.04 mm of maximum difference of R_80_. With range shifters, maximum difference of R_80_ was 0.23 mm. In‐air spot sizes for all energies were predicted within 10% tolerance (Figure 8b). The absolute doses at 2 cm WET depth were calculated within less than 2% when the energy was above 80 MeV; the predicted output at 69 MeV showed up to 3.3% difference from the measurement.

## 4 DISCUSSION

The commissioning of the Varian multi‐room ProBeam 360° PBS proton therapy system and the clinical implementation of the RayStation TPS were presented. The BP chamber, with 4.1 cm radius, which was used for the measurements was known to not account for charges in the low dose tail region that extends beyond the radius of the chamber. However, the version of RayStation used (12ASP1) does not recommend any post processing to account for the finite size of the detector. Rather the beam modeling process accounts for the finite size of the detector.

**FIGURE 9:**
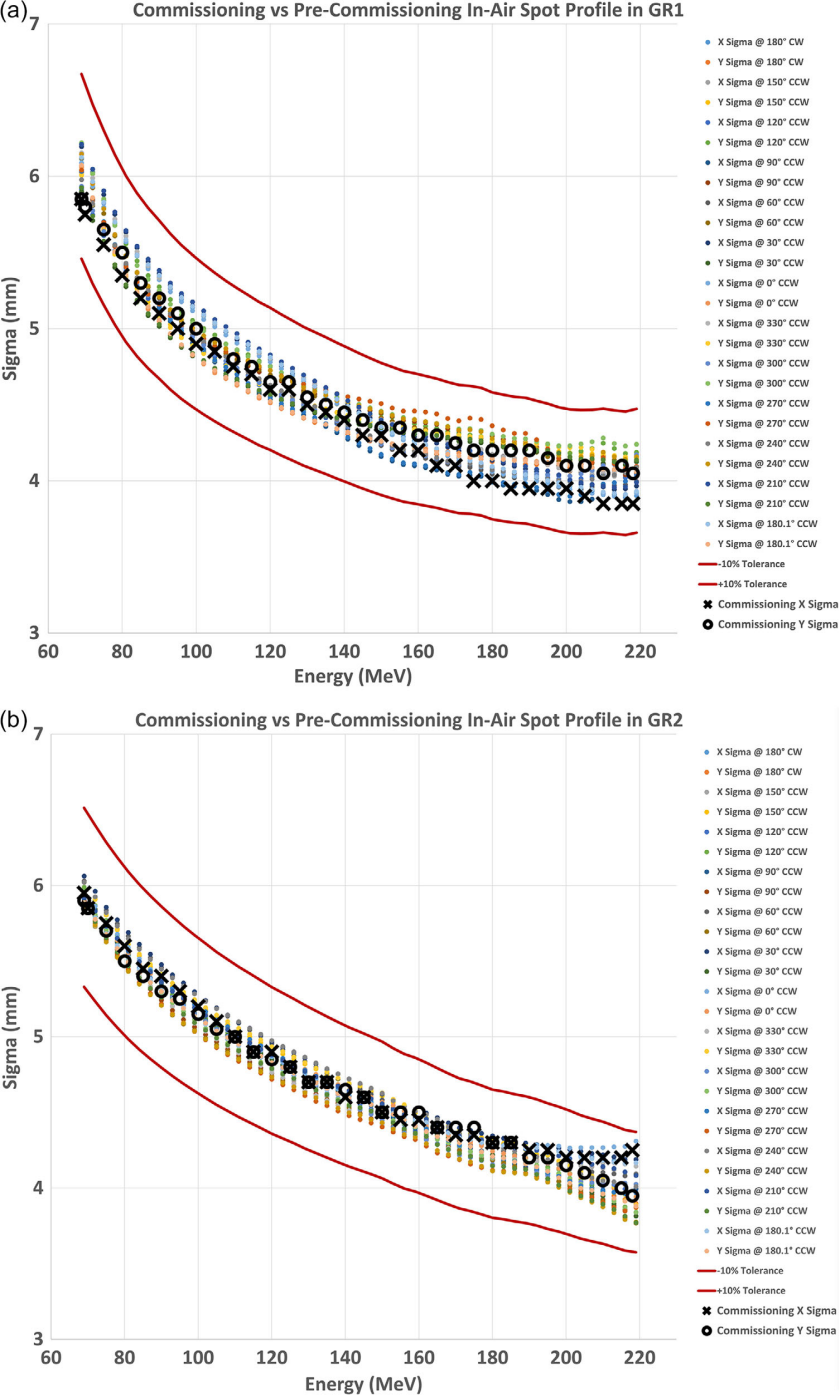
Pre‐commissioning in‐air spot sizes acquired with the vendor‐supplied detector at 30° increments are shown across gantry rotation for (a) GR1 and (b) GR2. Commissioning spot sizes at 0° and 90° are overlaid for comparison. Across all surveyed angles and energies, spot sizes are within the ±10% vendor tolerance, and the commissioning measurements align with the pre‐commissioning trends.

For TPS beam modeling we measured in‐air spot profiles at 0° and 90°, which capture the principal magnet‐sag orientations and meet the RayStation data requirements. To provide comprehensive context across gantry rotation, we incorporated pre‐commissioning measurements obtained during installation using the gantry mounted vendor's detector at 30° increments (Figure 9). While these pre‐commissioning data were acquired with a different detector and not used in TPS modeling, they independently demonstrate that spot sizes across all surveyed angles lie within the ±10% vendor tolerance and that our commissioning measurements at 0° and 90° lie on the same trend. Collectively, these results indicate that the gantry‐angle dependency is within specification, that 0° and 90° measurements are representative for TPS modeling, and that angles orthogonal to these (e.g., 270°) also remain within tolerance, thereby bracketing magnet sag‐related behavior despite the practical constraint at 180° in our commissioning setup. The largest difference in spot sizes (9.3%) between GR1 and GR2 occurs at the highest energy of 218 MeV but is still within the vender specifications. Since these profiles were measured in air, the impact of the difference in these spot sizes will be limited to surface and shallower dose. For these higher energies at depth, the difference will have a clinically negligible impact because spot size there depends on elastic scattering over a larger water equivalent path. Differences observed will smear out at depth inside the patient^13^.

Since the transport of protons through the range shifter is modeled in the RayStation dose engine, no beam modeling data measures were required. Only geometrical measures and material properties of the range shifters are defined in the beam modeling process.

An independent verification of dose to MU calibration was performed by irradiating thermoluminescent (TLD) dosimeters from the Imaging and Radiation Oncology Core (IROC, Houston, TX). The outputs in both GR1 and GR2 were verified within 1% before the clinical release of the TPS. The HU to RLSP conversion curves of our CT scanners are within 2σ values of all participating institutions monitored by IROC‐H. In order to credential the use of proton PBS delivery modality in National Cancer Institute‐sponsored clinical trials, the approval process by the IROC Houston has been completed as of May 2024. This includes various anthropomorphic phantoms, electronic data transfer, annual TLD output check, site visit and proton questionnaire completion.

In addition to validating essential beam parameters for TPS commissioning, some important beam characteristics were also validated. Beam isocentricity was periodically performed during the commissioning beam data acquirements using a cone‐shaped scintillation detector, XRV‐124 (Logos System Int'l, Scotts Valley, CA) to maintain the beam isocenter deviation relative to image guidance system within 1 mm. Gantry angle dependency of the calibrated output was validated within 1% at gantry angles of 0°, 90° and 270°. MU linearity was found to be within 0.5%. In order to routinely check all these clinical commissioning beam data and other parameters, periodic quality assurance protocol was established to fulfill the recommendation of American Association of Physicists in Medicine task group 224.^14^

Paired comparisons of beam parameters (absolute dose, in‐air spot size σ in x and y, and distal range R_80_) yielded very small *p*‐values (< 0.05), indicating that the mean GR2–GR1 differences are statistically distinguishable from zero given the high measurement precision and the number of energies sampled. Importantly, these differences are small in magnitude, and their 95% CI lie within pre‐specified clinical tolerances and equivalence margins (R_80_ ±1 mm, σ ±10%, dose ±1.5%), confirming that the rooms are beam‐matched for commissioning purposes. These findings support a common TPS beam model with room‑specific absolute dose calibration verified by independent IROC testing. In contrast, PSQA gamma pass rates showed *p*‐values > 0.05 (3%/3 mm: *p*  =  0.053; 3%/2 mm: *p*  =  0.146; 2%/2 mm: *p*  =  0.165), indicating no statistically significant room effect on clinical plan verification. Moreover, TOST equivalence with a ±2 pp margin was met for all criteria, ensuring an overall 95% confidence that any room‐to‐room difference in PSQA performance is clinically negligible. Note that statistical significance alone does not imply clinical relevance; therefore, equivalence testing was performed to confirm whether differences fall within predefined clinical tolerances, ensuring that both beam parameters and PSQA results are interpreted in terms of practical impact rather than purely statistical detectability. Together, these results support the use of a common TPS beam model for both rooms, with room‐specific absolute dose calibration verified independently.

## 5 CONCLUSION

The successful commissioning of the Varian multi‐room ProBeam 360° system at the Ohio State University has provided valuable insights into clinical readiness and operational performance of the system. The extensive validation process including beam characteristics and dosimetric accuracy confirmed that the system meets clinical requirements, with beam‐matching across multiple gantry rooms showing potential benefits in streamlining commissioning efforts and improving workflow efficiency. The data gathered from this process may help refine treatment planning models and offer useful reference points for upcoming installations. Additionally, these findings contribute to the broader understanding of beam characteristics in similar proton therapy systems, supporting ongoing efforts to optimize consistency across institutions.

## AUTHOR CONTRIBUTIONS

Eunsin Lee, Austin M Faught, Hyeri A Lee, Estelle Batin, and Ahmet Ayan contributed to design of the work, data acquisition, analysis and interpretation of data. Eunsin Lee, Austin M Faught and Hyeri A Lee drafted the manuscript, and Estelle Batin and Ahmet Ayan reviewed it. All authors approved the final version to be published and agree to be accountable for all aspects of the work in ensuring that questions related to the accuracy or integrity of any part of the work are appropriately investigated and resolved.

## CONFLICT OF INTEREST STATEMENT

The authors declare no conflicts of interest.

## Data Availability

The data that support the findings of this study are available from the corresponding author upon reasonable request.

## Footnotes

a The resolution of proton beam energy selection was verified down to 0.01 MeV during the acceptance test. 0.01 MeV energy difference corresponds to a tenth of submillimeter in WET, which is much smaller than measurement uncertainties from a typical water tank scan. This resolution is mainly determined by mechanical limit of degrader.

b Due to the constraint of the Lynx detector setup configuration with no support hardware, acquiring spot‐profiles at 180° was not practically feasible; this angle was therefore omitted from the commissioning dataset, with gantry‐angle constancy corroborated instead using the pre‐commissioning angle‐sweep measurements acquired at every 30°
